# Osteocyte RANKL is required for cortical bone loss with age and is induced by senescence

**DOI:** 10.1172/jci.insight.138815

**Published:** 2020-10-02

**Authors:** Ha-Neui Kim, Jinhu Xiong, Ryan S. MacLeod, Srividhya Iyer, Yuko Fujiwara, Keisha M. Cawley, Li Han, Yonghan He, Jeff D. Thostenson, Elisabeth Ferreira, Robert L. Jilka, Daohong Zhou, Maria Almeida, Charles A. O’Brien

**Affiliations:** 1 Center for Musculoskeletal Disease Research,; 2 Division of Endocrinology, Department of Internal Medicine, and; 3 Department of Orthopaedic Surgery, University of Arkansas for Medical Sciences, Little Rock, Arkansas, USA.; 4 Department of Pharmacodynamics, College of Pharmacy, University of Florida, Gainesville, Florida, USA.; 5 Department of Biostatistics, University of Arkansas for Medical Sciences, Little Rock, Arkansas, USA.; 6 Central Arkansas Veterans Healthcare System, Little Rock, Arkansas, USA.

**Keywords:** Bone Biology, Bone disease

## Abstract

In aging mice, osteoclast number increases in cortical bone but declines in trabecular bone, suggesting that different mechanisms underlie age-associated bone loss in these 2 compartments. Osteocytes produce the osteoclastogenic cytokine RANKL, encoded by *Tnfsf11*. *Tnfsf11* mRNA increases in cortical bone of aged mice, suggesting a mechanism underlying the bone loss. To address this possibility, we aged mice lacking RANKL in osteocytes. Whereas control mice lost cortical bone between 8 and 24 months of age, mice lacking RANKL in osteocytes gained cortical bone during this period. Mice of both genotypes lost trabecular bone with age. Osteoclasts increased with age in cortical bone of control mice but not in RANKL conditional knockout mice. Induction of cellular senescence increased RANKL production in murine and human cell culture models, suggesting an explanation for elevated RANKL levels with age. Overexpression of the senescence-associated transcription factor Gata4 stimulated *Tnfsf11* expression in cultured murine osteoblastic cells. Finally, elimination of senescent cells from aged mice using senolytic compounds reduced *Tnfsf11* mRNA in cortical bone. Our results demonstrate the requirement of osteocyte-derived RANKL for age-associated cortical bone loss and suggest that increased *Tnfsf11* expression with age results from accumulation of senescent cells in cortical bone.

## Abstract 2

Induction of senescence in osteoblast-like cells stimulates RANKL production, providing a possible explanation for the increased cortical bone resorption that occurs with age.

## Graphical abstract

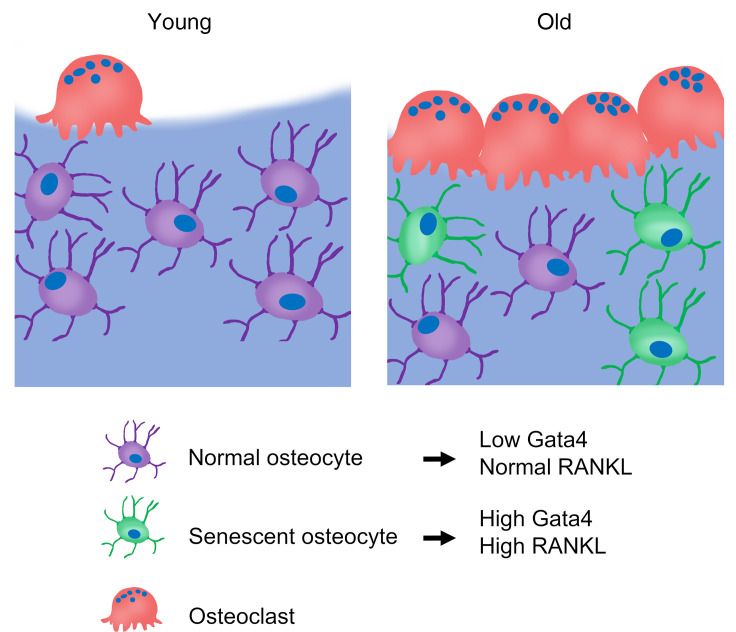

## Introduction

Both women and men lose bone mass with age (1). Women begin to lose bone before menopause, and men lose bone with age despite maintaining functional levels of sex steroids (2). Mice also exhibit age-associated bone loss, but this is not associated with sex steroid deficiency in either sex (3). Thus, although low estrogen accelerates the loss of bone in women, other mechanisms underlie age-associated bone loss in humans and mice.

Bone loss in humans occurs in both the trabecular and cortical compartments. Trabecular bone loss is characterized by trabecular thinning and an overall decrease in trabecular bone volume. Cortical loss is due to cortical thinning and increased cortical porosity. Similar structural changes occur in mice (4). We have shown that trabecular bone loss in mice is associated with a low rate of bone remodeling, whereas cortical thinning and porosity are associated with increased remodeling (4, 5). These different cellular changes suggest that different molecular mechanisms drive age-related bone loss in trabecular versus cortical bone.

The molecular mechanisms responsible for the increased osteoclast number in cortical bone with age are unclear. We have shown that production of the osteoclastogenic cytokine RANKL increases with age in murine cortical bone (4). Moreover, this change is associated with increased markers of cellular senescence in osteocyte-enriched cortical bone. Cellular senescence is a process in which cells stop dividing and initiate a gene expression pattern known as the senescence-associated secretory phenotype (SASP) (6–9). SASP factors reinforce growth arrest in an autocrine manner and alter the behavior of surrounding cells in a paracrine manner (10–12). Work performed with genetic models of senescent cell ablation or with drugs that eliminate senescent cells (senolytics) suggests that senescent cells contribute to skeletal aging (13). However, the identity of the senescent cells that contribute to skeletal aging, and the molecular mechanisms by which they may control bone remodeling, remain unknown.

Production of RANKL, which is encoded by the *Tnfsf11* gene, by osteocytes contributes to physiological bone resorption and some forms of pathological bone resorption, such as occurs during estrogen deficiency or hyperparathyroidism (14–17). However, the role of osteocyte-derived RANKL during aging is unknown, as are the mechanisms responsible for increased *Tnfsf11* expression with age. Here, we have investigated whether osteocyte-derived RANKL is required for age-related bone loss and whether senescence of osteocytes may stimulate RANKL production in this cell type.

## Results

### Mice lacking RANKL in osteocytes do not lose cortical bone with age.

**Figure 1:**
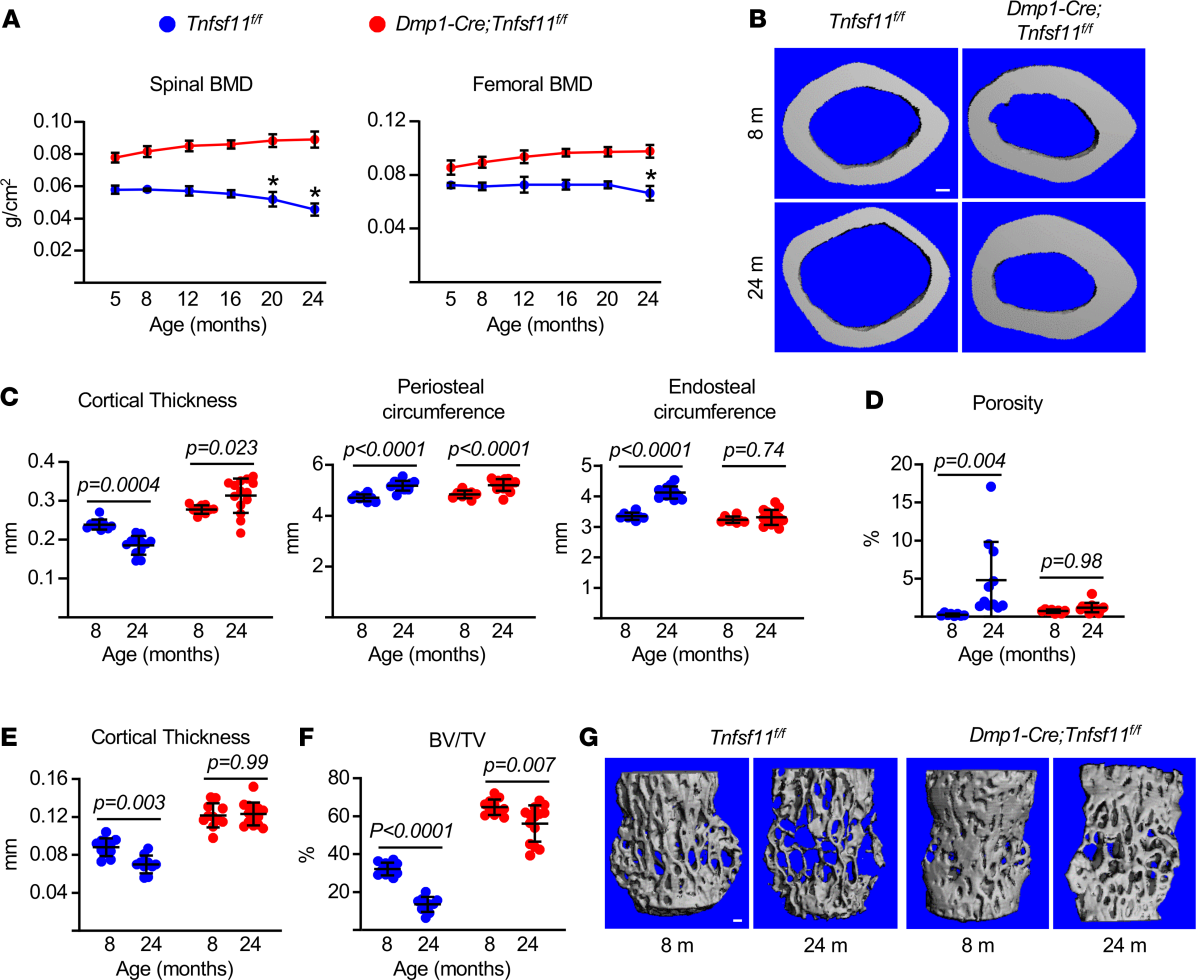
Age-related cortical bone loss requires osteocyte RANKL. A cohort of female *Dmp1-Cre;Tnfsf11^fl/fl^* and *Tnfsf11^fl/fl^* littermate controls were aged to 24 months. Another cohort of female mice of the same genotypes euthanized at 8 months of age served as young controls. (**A**) Sequential BMD measurements in the aging cohort by dual-energy x-ray absorptiometry (*n*= 8/group). (**B**) Representative micro-CT images of femoral midshaft cortical bone (scale bar: 100 μm). (**C**) Cortical bone measurements at the midshaft and (**D**) cortical porosity at the distal metaphysis of the femur by micro-CT (*n* = 10–14/group). (**E**) Cortical thickness and (**F**) trabecular bone volume (BV/TV) in L4 vertebra by micro-CT (*n* = 10–14/group). (**G**) Representative micro-CT images of trabecular bone in L4 (scale bar: 100 μm). Lines and error bars represent mean ± SD. (**A**) *P < 0.001 versus 5 months of age in the same genotype by repeated-measures ANOVA; ANOVA also showed that BMD was different between genotypes at each age in both the spine and femur (not indicated in graphs). (**C**–**F**) *P* values were determined using 2-way ANOVA.

To determine whether RANKL produced by osteocytes is required for age-related bone loss, we generated female mice lacking the *Tnfsf11* gene in this cell type using *Dmp1-Cre*–transgenic mice (18). Littermates homozygous for the conditional allele but lacking the *Dmp1-Cre* transgene were used as controls. Serial analysis of bone mineral density (BMD) revealed that control mice exhibited age-related bone loss in the spine beginning at 20 months of age and in the femur at 24 months (Figure 1A). In contrast, conditional knockout mice, which had higher bone mass than controls at all ages, did not exhibit loss of BMD with age in the spine or femur (Figure 1A).

The bones of 24-month-old mice were then compared with bones from 8-month-old mice of the same genotype by micro-CT analysis. As expected, control mice had lower femoral cortical thickness at 24 versus 8 months of age (Figure 1, B and C). Conversely, old mice lacking RANKL in osteocytes had thicker cortical bone than young conditional knockout mice (Figure 1, B and C). Mice of both genotypes exhibited increased periosteal expansion with age, but the increase in endosteal circumference that occurred in control mice with age did not occur in mice lacking RANKL in osteocytes (Figure 1C). Similarly, the increase in cortical porosity that developed with age in control mice did not develop in conditional knockout mice (Figure 1D). Vertebral cortical bone thickness was also reduced with age in control mice but not in conditional knockout mice (Figure 1E). However, in contrast to cortical bone, mice lacking RANKL in osteocytes lost trabecular bone with age (14%), albeit to a lesser extent than control mice (56%) (Figure 1, F and G). These results demonstrate that RANKL produced by osteocytes is required for the loss of cortical bone with age but that loss of trabecular bone still occurs in the absence of osteocyte RANKL.

**Figure 2:**
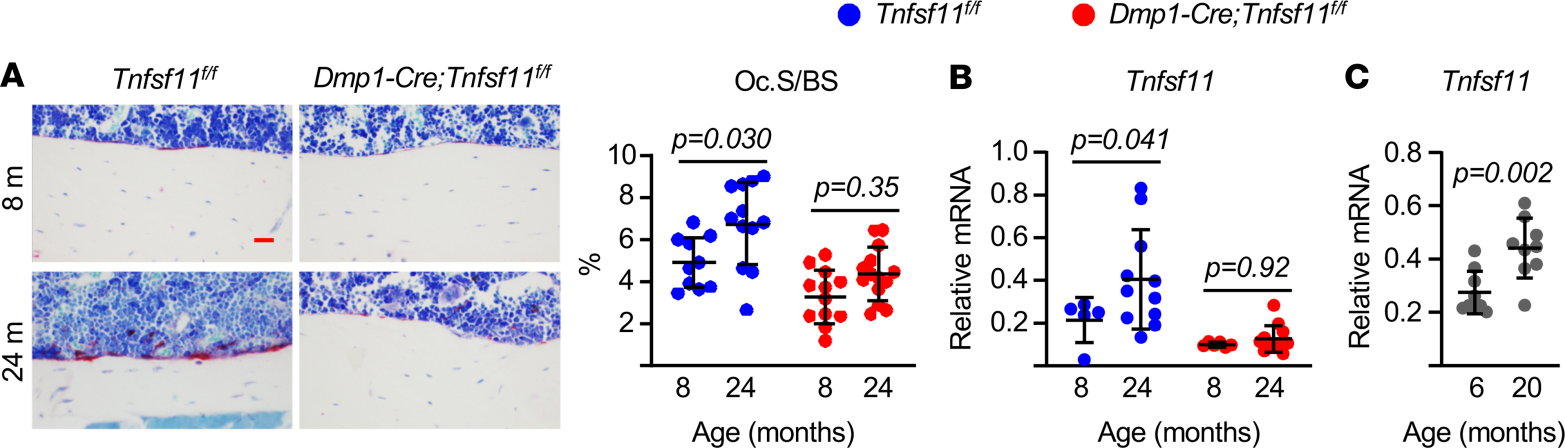
Elevated RANKL and resorption in aged cortical bone. (**A**) Representative photomicrographs of nondecalcified femur sections stained for TRAPase activity (red) (scale bar: 100 μm) and quantitation of osteoclast surface per bone surface (Oc.S/BS) measured in longitudinal nondecalcified femur sections of 8- and 24-month-old female *Dmp1-Cre;Tnfsf11^fl/fl^* mice and *Tnfsf11^fl/fl^* littermate controls (*n* = 10–14/group). (**B**) mRNA levels of *Tnfsf11* by qPCR of RNA from osteocyte-enriched bone shafts of 8- and 24-month-old female *Dmp1-Cre;Tnfsf11^fl/fl^* mice and *Tnfsf11^fl/fl^* littermate controls (*n* = 5–12/group). (**C**) *Tnfsf11* mRNA measured in RNA from osteocyte-enriched bone shafts of 6- and 20-month-old male C57BL/6 mice (*n* = 9/group). Lines and error bars represent mean ± SD; *P* values by 2-way ANOVA (**A** and **B**) or *t* test (**C**).

The preservation of endocortical bone in the 24-month-old conditional knockout mice suggested that they were protected from the increase in endocortical bone resorption that occurs with age (4). Histomorphometric quantification of osteoclasts at this site confirmed that the endocortical bone surface covered by osteoclasts increased with age in control mice but not in conditional knockout mice (Figure 2A). We have shown previously that expression of *Tnfsf11* increases with age in osteocyte-enriched cortical bone (4). We observed a similar increase in the control mice of the present study, but this did not occur in the conditional knockout mice, demonstrating that the increase in *Tnfsf11* mRNA observed in aged cortical bone originates from osteocytes (Figure 2B). In both the present study and our previous study we measured *Tnfsf11* mRNA abundance in female mice (4). Analysis of cortical bone RNA from male WT C57BL/6 mice revealed increased *Tnfsf11* mRNA with age as well (Figure 2C). Taken together, these results suggest that, as osteocytes age in cortical bone, they begin to express higher levels of *Tnfsf11* and this is required for the increase in osteoclast formation at the endocortical surface and subsequent bone loss. These findings raise the question of what mechanisms might be responsible for the age-related increase in *Tnfsf11* expression by osteocytes.

### Induction of senescence is sufficient to increase RANKL production.

The gene expression profile of cortical bone from aged mice, which shows increased cell cycle inhibitors and inflammatory cytokines, suggests the presence of senescent cells (4). Since senescence is associated with increased production of inflammatory proteins, many of which can stimulate *Tnfsf11* expression, these findings suggested that an increase in osteocyte senescence might contribute to the age-related increase in RANKL production in cortical bone. To address this possibility, we determined whether induction of senescence in cells of the osteoblast lineage is sufficient to stimulate *Tnfsf11* expression.

**Figure 3:**
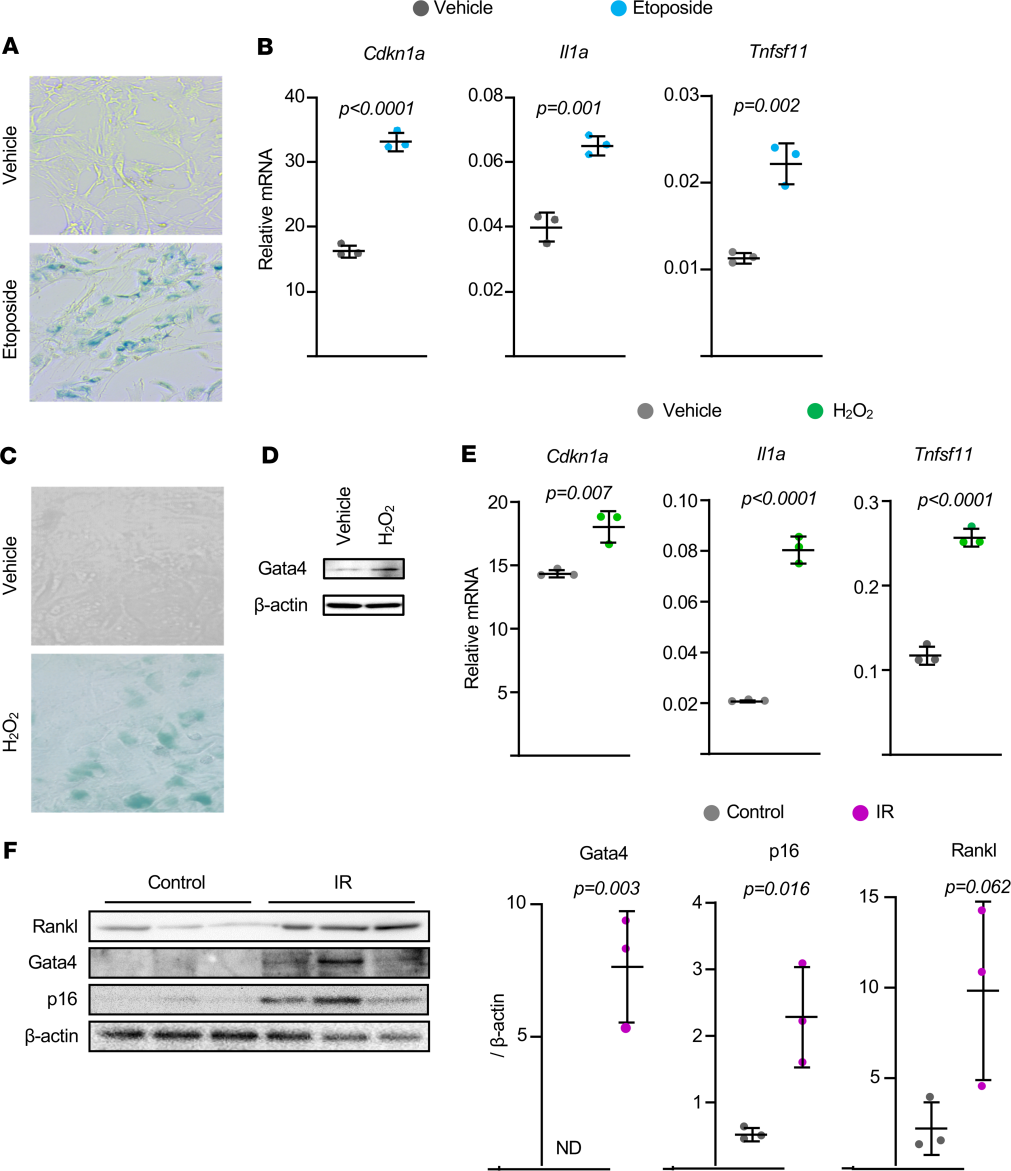
Senescence stimulates RANKL production in murine cells. (**A**–**E**) Bone marrow stromal cells isolated from 6-month-old female mice and cultured with (**A** and **B**) 10 μM etoposide or (**C**–**E**) 25 μM H_2_O_2_ for 3.5 days. (**A** and **C**) Representative photomicrographs of SA–β-gal staining of stromal cell cultures. Original magnification, ×100. (**B** and **E**) Gene expression by quantitative RT-PCR (triplicate cultures). (**D**) Protein levels by Western blot. (**F**) Osteocyte-enriched bone shafts isolated from 6-month-old female mice and cultured for 5.5 days after ionizing radiation (IR) exposure to 10 Gy. Protein levels by Western blot (left) and band density quantification (right) from 3 independent mice. Line and error bars represent mean ± SD; *P* values by 2-tailed unpaired *t* test.

The first approach we used to cause senescence was induction of DNA damage using the topoisomerase inhibitor etoposide (19). Murine bone marrow–derived stromal cells exposed to etoposide displayed markers of senescence, including β-Gal activity and elevated expression of the cell cycle inhibitor p21, encoded by *Cdkn1a* (Figure 3, A and B). Etoposide also increased production of IL-1α, a cytokine component of the SASP in many cell types (Figure 3B). Importantly, these changes were associated with increased abundance of *Tnfsf11* mRNA (Figure 3B). We next induced senescence by culturing bone marrow stromal cells with H_2_O_2_ (Figure 3, C–E). In addition to the increase in β-Gal and p21, we also observed an increase in the abundance of *Gata4*, a transcription factor known to increase expression of SASP genes (Figure 3D) (20). Again, these changes were accompanied by increased *Tnfsf11* mRNA (Figure 3E). To determine whether induction of senescence can stimulate RANKL production in osteocytes, we irradiated osteocyte-enriched cortical bone cultures. Irradiation increased the amount of Gata4 protein as well as the abundance of the cell cycle inhibitor p16, encoded by *Cdkn2a* (Figure 3F), which is induced by senescence in many cell types (20). Importantly, these changes were also associated with increased RANKL protein levels in cortical bone (Figure 3F).

**Figure 4:**
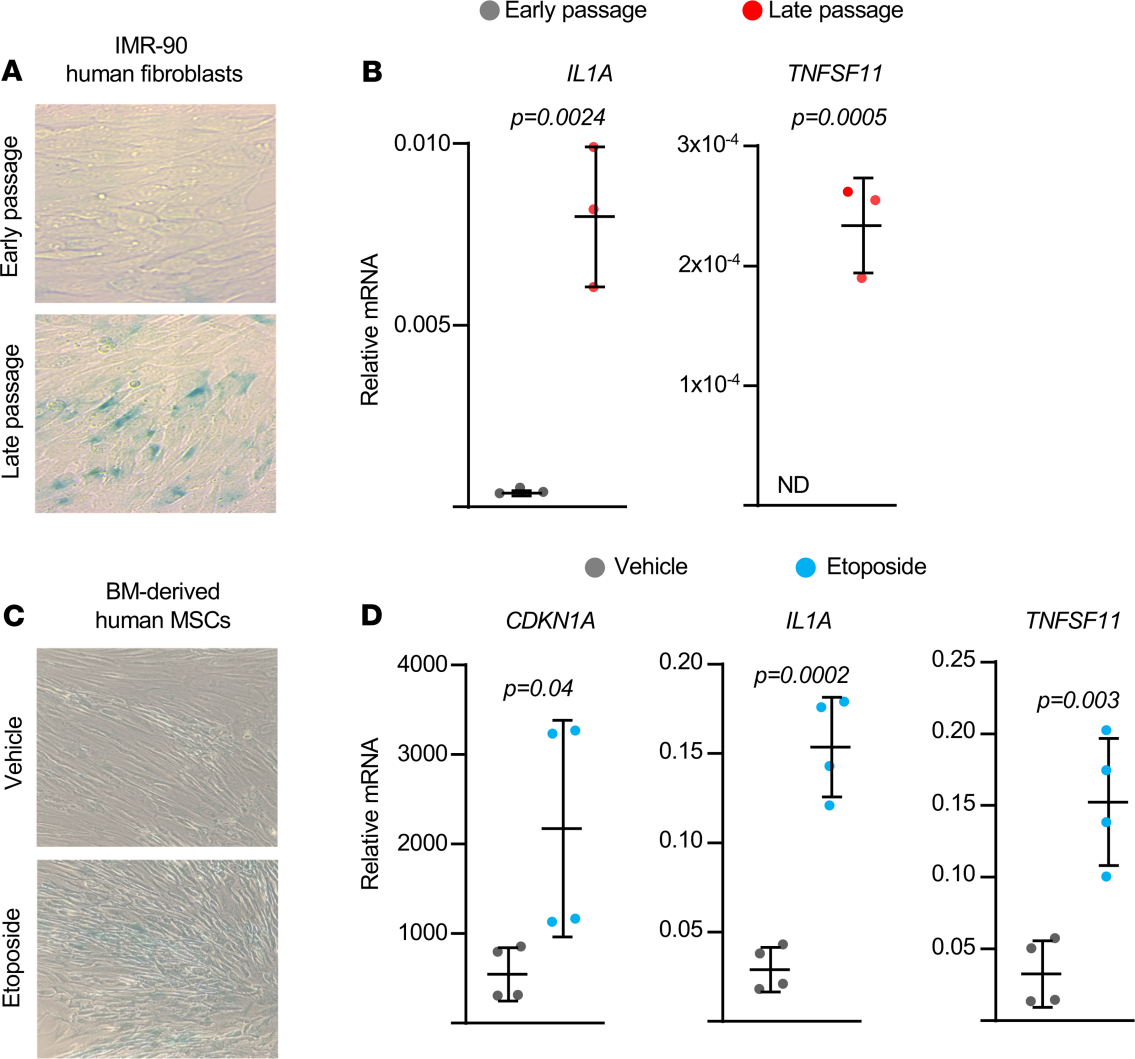
Senescence stimulates RANKL production in human cells. (**A**) Representative photomicrographs of SA–β-gal–stained cultures and (**B**) mRNA levels by qRT-PCR in nonsenescent and senescent IMR-90 cells induced by replicative exhaustion (triplicate cultures). Original magnification, ×100. (**C**) Representative photomicrographs of SA–β-gal–stained cultures and (**D**) mRNA levels in human mesenchymal stromal cells isolated from 29 year-old and 33 year-old male donors, cultured with 50 μM etoposide for 4 days. Original magnification, ×100. Line and error bars represent mean ± SD; *P* values by 2-tailed unpaired *t* test.

We also determined whether senescence stimulates RANKL production in human IMR-90 fibroblasts, which are used extensively to study replicative senescence (21). As expected, late-passage cells exhibited increased senescence-associated β-Gal activity (Figure 4A). Senescent cells also exhibited high levels of *IL1*A mRNA (Figure 4B). While *TNFSF11* mRNA was undetectable in early-passage IMR-90 cells, it was easily detected after induction of senescence in late-passage cells (Figure 4B). To confirm these results, we induced senescence in bone marrow–derived human mesenchymal stromal cells using etoposide and found that the drug increased senescence-associated β-Gal activity and expression of both *IL1A* and *TNFSF11* (Figure 4, C and D). These results suggest that induction of senescence is sufficient to stimulate RANKL production in both murine and human cells.

### Overexpression of Gata4 causes senescence and increases RANKL.

**Figure 5:**
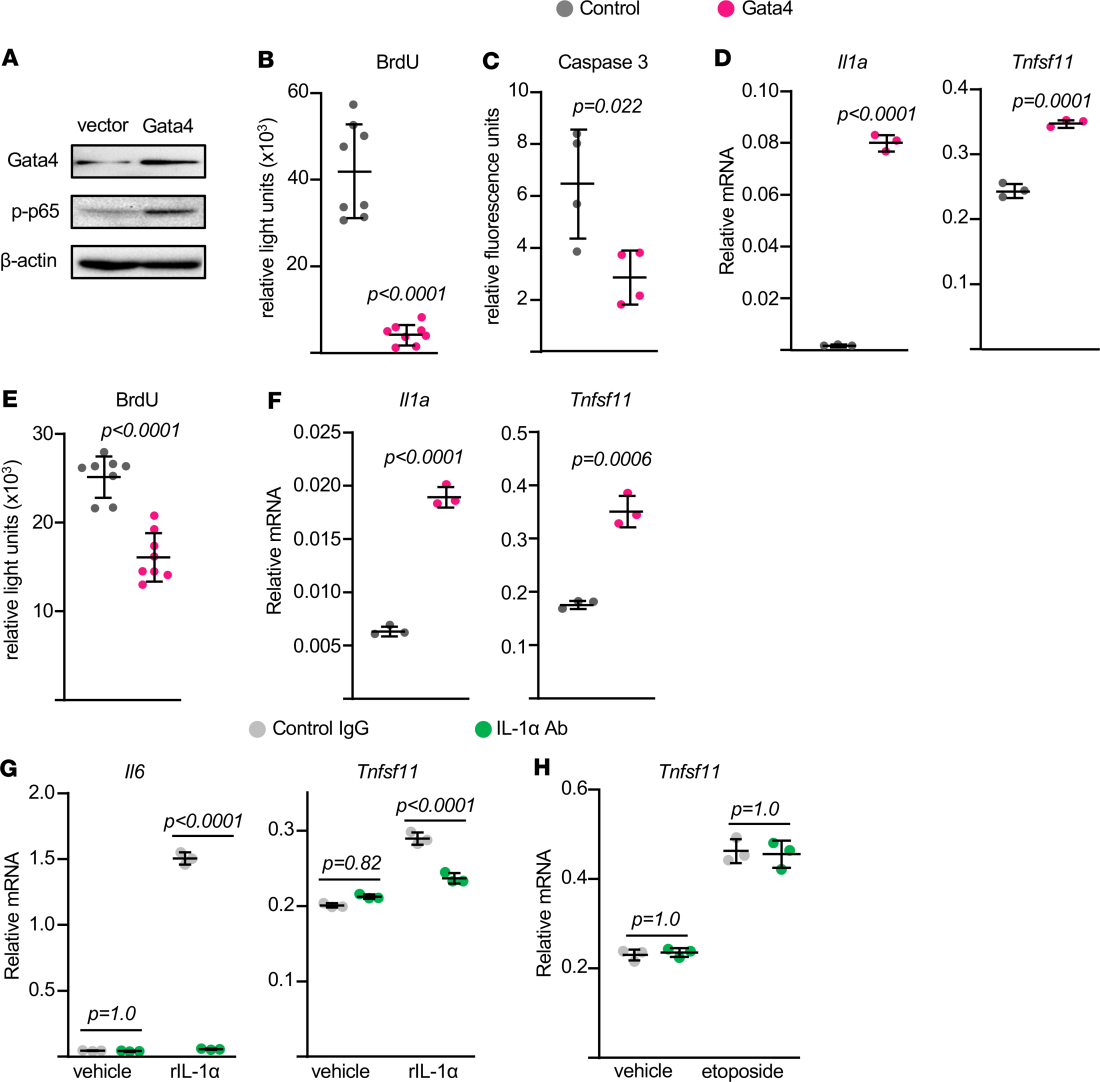
Gata4 overexpression stimulates RANKL production. Newborn calvaria cells (**A–D**) or bone marrow–derived stromal cells (**E** and **F**) were transduced with retroviruses expressing empty vector or Gata4 and cultured for 24 hours (**A–D**) or 72 hours (**E** and **F**). (**A**) Protein levels by Western blot. (**B** and **E**) Proliferation by BrdU labeling (*n* = 8/group). (**C**) Apoptosis by caspase-3 activity (*n* = 4/group). (**D** and **F**) mRNA levels by qRT-PCR (triplicate cultures). (**G** and **H**) Bone marrow–derived stromal cells were pretreated with control IgG or neutralizing anti–IL-1a antibody (5 μg/mL) for 30 minutes, followed by (**G**) recombinant IL-1a (10 ng/mL) or (**H**) etoposide (10 μM) for 24 hours. mRNA levels by qRT-PCR (triplicate cultures). Line and error bars represent mean ± SD; *P* values by 2-tailed unpaired *t* test (**B**–**F**) or 2-way ANOVA (**G** and **H**).

To explore the mechanisms by which senescence stimulates RANKL production in cells of the osteoblast lineage, we overexpressed the transcription factor Gata4. Previous studies have demonstrated that increased abundance of Gata4 in senescent cells underlies stimulation of genes encoding components of the SASP (20). Overexpression of Gata4 in calvaria-derived osteoblastic cells increased phosphorylation of p65, reduced proliferation, and reduced apoptosis (Figure 5, A–C), suggesting that high levels of Gata4 are sufficient to induce at least some of the cellular changes previously shown to be associated with senescence (20). These changes were associated with increased production of both *Il1a* and *Tnfsf11* mRNA (Figure 5D). Overexpression of Gata4 in bone marrow–derived osteoblastic cells also concomitantly reduced proliferation and increased production of these cytokines (Figure 5, E and F).

In some cell types, stimulation of IL-1α mediates at least in part the induction of SASP by GATA4 (20). Because IL-1α can stimulate *Tnfsf11* expression, we examined whether IL-1α was a mediator of the effects of senescence on RANKL. As shown earlier (22, 23), recombinant IL-1α stimulated the levels of *Il6* and *Tnfsf11* mRNA in bone marrow–derived osteoblastic cells (Figure 5G). This effect was attenuated by addition of an IL-1α antibody to the cultures, confirming the ability of the antibody to block IL-1α action. However, the IL-1α antibody had no effect on the etoposide-induced increase in *Tnfsf11* mRNA (Figure 5H). These results suggest that stimulation of RANKL is not secondary to the increase in IL-1α during osteoblastic cell senescence.

### Administration of senolytics to old mice decreases RANKL.

**Figure 6:**
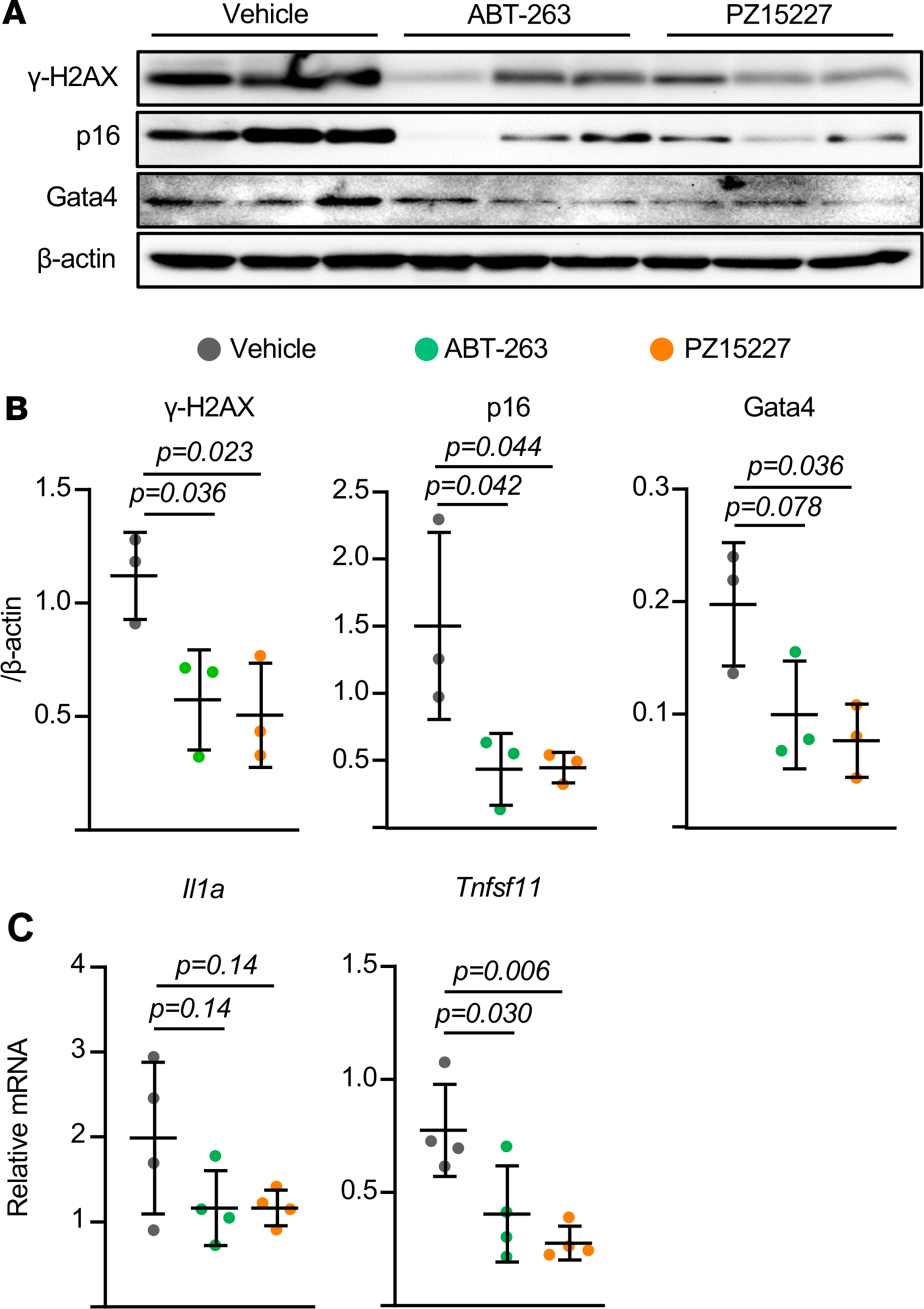
Removal of senescent cells reduces RANKL levels in aged bone. Osteocyte-enriched bone shafts were isolated from 24-month-old female mice that had received vehicle, ABT-263, or PZ15227 by daily i.p. injections for 5 days. (**A**) Protein levels by Western blot and (**B**) band density quantification from 3 independent mice. (**C**) mRNA levels by qRT-PCR from 4 independent mice. Lines and error bars represent mean ± SD; *P* values by 1-way ANOVA.

To determine whether senescent cells contribute to the increased RANKL production in the bone of aged mice, we sought to eliminate senescent cells from aged mice using senolytics and examine the effect on gene expression. The bcl-2 inhibitor ABT263 or a less toxic and more potent analog of ABT263, known as PZ15227 (24), were administered to 24-month-old female mice for 5 days. Measurement of the DNA damage marker H2AX in osteocyte-enriched bone revealed reduced levels of DNA damage in the bones of mice treated with either of the senolytic drugs (Figure 6, A and B). Moreover, the senescent marker proteins p16 and Gata4 were also reduced by senolytic administration (Figure 6, A and B). Together, these results suggest effective killing of senescent cells in the bones of the aged mice. Importantly, removal of the senescent cells was associated with reduced abundance of both *Il1a* and *Tnfsf11* mRNA (Figure 6C). These results support the conclusion that senescent cells in the bones of aged mice are a source of RANKL or stimulate other cells in bone to produce this cytokine.

## Discussion

The structural integrity of cortical bone decreases with age due to loss of endocortical bone and an increase in intracortical porosity (25). Conceptually, these changes could result from increased bone resorption, decreased bone formation, or both. Herein, we provide evidence that increased bone resorption driven by increased production of RANKL by osteocytes is essential for the age-associated loss of cortical bone. Moreover, our results suggest that, if bone formation in cortical bone declines with age, the extent of this decline is not sufficient to cause bone loss in the absence of increased resorption.

In mammals, cortical thickness remains relative constant from early adulthood until the onset of age-associated changes, which in C57BL/6J mice is at approximately 1 year of age (26). More specifically, postgrowth expansion of the medullary cavity accelerates after 1 year of age in this strain and cortical porosity begins to develop (26). Therefore, the molecular and cellular changes responsible for age-associated bone loss begin to manifest themselves at this time. The results of the present study suggest that accumulation of senescent osteocytes, and a subsequent increase in RANKL production, are key mechanisms responsible for the increase in endocortical and intracortical resorption.

It has been suggested that the periosteal expansion that occurs with advancing age is a compensatory response to endocortical bone loss in an attempt to maintain resistance to bending (27). More specifically, the rapid loss of endocortical bone at menopause, and the subsequent increase in mechanical stress, has been proposed to stimulate periosteal bone formation (28). However, recent findings in women transitioning through menopause do not support this idea, since the women with greater bone loss experienced lower levels of periosteal apposition (29). Our finding that old mice lacking RANKL in osteocytes exhibit normal periosteal expansion, even though they did not lose endosteal bone, also supports the idea that periosteal expansion is unrelated to endocortical bone loss.

Several lines of evidence presented in our study support the idea that accumulation of senescent cells contributes to the increase in RANKL expression with age. First, induction of cellular senescence via DNA damage stimulated *Tnfsf11* expression in multiple mesenchymal cell models. Second, overexpression of *Gata4* in primary osteoblastic cells induced markers of senescence as well as RANKL production. Third, elimination of senescent cells in aged mice reduced *Tnfsf11* mRNA abundance in bone tissue. It remains unclear, however, whether increased expression of *Tnfsf11* by osteocytes is driven by senescence of osteocytes or instead by paracrine factors produced by senescent cells other than osteocytes.

Although we did not examine the long-term effects of senolytic administration in the current study, Farr and colleagues have reported that 4 months of senolytic administration to aged mice reduces osteoclast number at the endocortical surface and increases cortical thickness (13). These investigators obtained similar results using the INK-ATTAC mouse model, in which cells expressing high levels of a p16-based transgene are eliminated by drug-induced activation of caspase-8 (13). In previous studies, we have also used a genetic approach to eliminate senescent cells from aged mice. Specifically, we used p16-3MR mice, in which cells expressing a different p16-based transgene are eliminated by addition of ganciclovir (30). With this model, we found that elimination of senescent cells from the age of 1 to 2 years had no discernible effects on cortical thickness (30). Unexpectedly, the genetic approach that we used did not eliminate senescent osteocytes, whereas they were reduced in the INK-ATTAC mice. This difference may explain the different effects on cortical bone between the two studies. Nevertheless, the studies of Farr and colleagues, together with our current findings, are consistent with the idea that increased production of RANKL by senescent osteocytes is responsible for the increase in endocortical osteoclasts and the thinning of cortical bone with old age.

Our results also demonstrate that Gata4 accumulation is a common event in senescent osteoblastic cells and osteocytes and occurs in response to different senescence-inducing stimuli. This is in line with our previous findings that Gata4 levels increase in osteoprogenitors and osteocyte-enriched bone from old mice (4, 31). Likewise, Gata4 levels increase in the liver and brain during physiological aging in both humans and mice and in mice treated with senescence-inducing stimuli (20). In postnatal, nonsenescent cells, Gata4 levels are low due to degradation by p62-mediated selective autophagy (20). When senescence occurs, this selective autophagy is suppressed, leading to Gata4 accumulation. Gata4 in turn stimulates production of IL-1α, which stimulates NF-κB and the SASP (20). Here, we show that an increase in Gata4 is sufficient to stimulate production of both IL-1α and RANKL. Khalid and coworkers have suggested that Gata4 binds to the *Tnfsf11* promoter and represses *Tnfsf11* expression (32). A reason for the difference between this previous study and the results presented here is not clear. It should be noted, however, that in the study by Khalid et al., overexpression of Gata4 stimulated, rather than suppressed, a *Tnfsf11* promoter construct (32), a finding that is consistent with our results. We also found that in osteoblastic cells the increase in RANKL levels by senescence stimuli is independent of IL-1α. Be that as it may, further genetic studies will be required to determine whether Gata4 mediates the increased production of RANKL by osteocytes with age and whether this occurs by direct regulation of the *Tnfsf11* gene or indirectly via intermediate factors, other than IL-1α.

The finding that mice lacking RANKL in osteocytes lose trabecular but not cortical bone mass with age strongly supports the contention that different aging mechanisms underlie the loss of bone in these 2 compartments. Our results are also in line with previous evidence that in old age osteoclast number increases at the endocortical surface but declines in trabecular bone (4, 5). While there is evidence for the accumulation of senescent osteocytes in cortical bone with old age, it remains unknown whether senescent osteocytes accumulate in trabecular bone. Therefore, a potential explanation for the difference in osteoclast number between the 2 compartments in aged mice may be that senescent osteocytes do not accumulate in trabecular bone. Determination of osteocyte life span in cortical versus trabecular bone will be required to address this idea.

As in other tissues, the molecular mechanisms responsible for skeletal changes with age are likely multifactorial. Nonetheless, our study identifies key aspects of cortical bone loss that differ from mechanisms underlying loss of trabecular bone, strongly supporting the contention that aging mechanisms intrinsic to bone cells, rather than changes in circulating factors or physical activity, underlie skeletal aging. Continued identification of these cell-intrinsic mechanisms may guide development of therapies to preserve skeletal assets during aging.

## Methods

### Animals.

To disrupt *Tnfsf11* in mature osteoblasts and osteocytes, *Tnfsf11^fl/fl^* mice (14) were crossed with mice harboring a *Dmp1-Cre* transgene (18). Female mice used in the aging experiment were littermates generated by crossing *Tnfsf11^fl/fl^* mice with *Dmp1-Cre;Tnfsf11^fl/fl^* mice. Offspring were genotyped by PCR using the following primer sequences: Cre-for, 5′-GCGGTCTGGCAGTAAAAACTATC-3′; Cre-rev, 5′-GTGAAACAGCATTGCTGTCACTT-3′ (product size, 102 bp); RANKLflox-for, 5′-CTGGGAGCGCAGGTTAAATA-3′; RANKLflox-rev, 5′-GCCAATAATTAAAATACTGCAGGAAA-3′ (product size, 108 bp [WT] and 251 bp [floxed allele]). At 8 months of age, the mice were switched to Teklab diet 2014, containing 14% protein and 4% fat and acidified water ad libitum, to prevent excessive weight gain, and euthanized at 24 months of age. Eight-month-old female mice of the same genotypes from a previous experiment were used as young adult controls (16). To test the effects of senolytic drugs, 24-month-old female C57BL/6 mice (National Institute on Aging–supported colonies maintained by Charles River Laboratories) were randomly assigned to one of the treatment groups and received i.p. injections of vehicle (0.1 mL/mouse, *n* = 4 mice), ABT263 (41 μmol/kg or 40 mg/kg, *n* = 4 mice), or PZ15227 (41 μmol/kg or 61 mg/kg, *n* = 4 mice) daily for 5 days. ABT263 and PZ15227 were formulated in 50% Phosal 50 PG (American Lecithin Company), 45% MIGLYOL 810 N (IOI Oleochemical), and 5% polysorbate 80 (Spectrum Chemical).

### Dual-energy x-ray absorptiometry and micro-CT.

Dual-energy x-ray absorptiometry was performed using a PIXImus densitometer (GE Lunar) on mice sedated with 2% isoflurane and data analyzed as previously described (14). Scans of the entire left femur or lumbar spine were used for measurement of BMD. Bone architecture was determined on dissected femora and lumbar vertebra (L4) cleaned of adherent tissue, fixed in Millonig’s phosphate buffer (Leica Biosystems), and stored in 100% ethanol. Bones were scanned with a MicroCT40 (Scanco Medical) at medium resolution (12 μm isotropic voxel size) for quantitative determinations. For the latter, a Gaussian filter (sigma = 0.8, support = 1) was applied. Scanco Eval Program v.6.0 was used for measuring bone volume. Scan settings included x-ray tube potential (55 kVp), x-ray intensity (145 μA), and integration time (220 ms). Nomenclature conforms to recommendations of the American Society for Bone and Mineral Research (33). Femora were scanned from the femoral head to the beginning of the distal growth plate. Cortical dimensions were determined at the diaphysis (18 slices, midpoint of the bone length as determined in scout view), and cortical porosity was measured at the metaphysis, starting 8–10 slices away from the growth plate so as to avoid the growth plate, and proceeding proximally for 151 slices, to obtain cross-sectional images drawn to exclude trabecular elements. Cortical bone was measured at a threshold of 200 mg/cm^3^. Trabecular analyses were performed on contours of the cross-sectional images drawn to exclude cortical bone and were measured at a threshold of 220 mg/cm^3^. Trabecular architecture was determined using sphere-filling distance transformation indices without assumptions about the bone shape as a rod or plate. The fourth lumbar vertebra (L4) was scanned from the rostral growth plate to the caudal growth plate to obtain 233 slices. BV/TV in the vertebra was determined using 100 slices (1.2 mm) of the anterior (ventral) vertebral body immediately inferior (caudal) to the superior (cranial) growth plate. Trabecular bone analyses were performed on contours of cross-sectional images, drawn to exclude cortical bone, as described for femoral trabecular bone. Vertebral cortical bone thickness was determined on the ventral cortical wall using contours of cross-sectional images, drawn to exclude trabecular bone, as described for femoral cortical bone.

### Bone histology.

Femurs were fixed for 24 hours in Millonig’s 10% formalin followed by dehydration, embedding in methyl methacrylate, and cutting of 5 μm longitudinal sections. The sections were stained for tartrate-resistant acid phosphatase (TRAP) activity using napthol AS-MX (MilliporeSigma) and Fast Red TR salt (MilliporeSigma) and counterstained with toluidine blue (MilliporeSigma). The number and the surface of TRAP-positive cells on both of the endosteal surfaces of longitudinal sections (osteoclast number and surface) were measured using an Olympus BX53 microscope and Olympus DP73 camera (Olympus America Inc.) interfaced with a digitizer tablet with Osteomeasure software version 4.1.0.2 (OsteoMetrics Inc.). Histomorphometry measurements were made in a blinded fashion. The terminology used in this study has been recommended by the Histomorphometry Nomenclature Committee of the American Society for Bone and Mineral Research (34).

### Cell culture.

Total bone marrow cells were obtained by flushing the tibiae and femora. Cells from 3- to 6-month-old C57BL/6 mice were pooled and cultured in α-MEM complete media containing 20% FBS, 1% penicillin and streptomycin (PSG), and 50 μg/mL ascorbic acid (MilliporeSigma) in 10 cm culture dishes for 7 days. The medium was replaced every 3 days. Adherent bone marrow stromal cells were replated in 12-well tissue culture plates at 0.2 × 10^6^ cells per well with 10% FBS, 1% PSG, 50 μg/mL ascorbic acid, and 10 mM β-glycerophosphate (MilliporeSigma) for 3 to 5 days to perform qPCR assays and Western blotting. To induce cellular senescence, the stromal cells were treated with 10 μM etoposide (MilliporeSigma) or 25 μM H_2_O_2_ (MilliporeSigma) for 3.5 days and used for subsequent assays. Recombinant IL-1α (400-ML-005) and neutralizing anti–IL-1α antibody (AB-400-NA) were obtained from R&D Systems Inc. To induce replicative senescence in human fibroblasts, IMR-90 cells (ATCC, CCL-186) were subcultured until they stopped dividing and became permanently growth arrested or senescent (about 37 passages). Low-passage IMR-90 cells (<25 passages) were used as nonsenescent cells. Bone marrow–derived human mesenchymal stromal cells (Lonza Inc.) from healthy donors (male, 29 and 33 years old) were seeded at 5000 cells/cm^2^ in DMEM-low glucose + 10%FBS and treated with 50 μM etoposide for 4 days. Cells were washed with 1× PBS and used for either senescence-associated β-Gal staining or RNA preparation.

### Organ culture.

To induce cellular senescence by irradiation, osteocyte-enriched cortical bone shafts were exposed to 10 Gy of irradiation as described before (31). Briefly, bone shafts were prepared from 6-month-old C57BL/6 female mice by removing the ends, flushing the bone marrow by centrifugation, and removing surface cells by scraping with a scalpel. Bone fragments from 3 mice were pooled and cultured in α-MEM complete media containing 10% (v/v) FBS and 1% PSG in 12-well tissue culture plates for 5.5 days or 7 days. Medium was replaced every 3 days. The cultured bone shafts were washed with 1× PBS 2 times, immediately cryopreserved in liquid nitrogen, and used for subsequent assays.

### SA–β-Gal staining.

Detection of SA–β-Gal activity was performed using a Senescence β-Galactosidase staining kit (Cell Signaling Technology) according to the manufacturer’s instructions. Briefly, cells were fixed with 2% formaldehyde/0.2% glutaraldehyde for 5 minutes at room temperature. After incubation with SA–β-Gal staining solution (1 mg/mL 5-bromo-4-chloro-3-indolyl-β-D-galactoside, 40 mM citric acid/sodium phosphate [pH 6.0], 5 mM potassium ferrocyanide, 5 mM potassium ferricyanide, 150 mM NaCl, 2 mM MgCl_2_) for 12 hours (bone marrow stromal cells) or 12–16 hours (IMR-90 cells and human mesenchymal stromal cells), senescent cells were identified as blue-stained cells by bright-field microscopy.

### RNA purification and gene expression.

Tibial or femoral cortical bone was prepared by removing the distal and proximal ends and removing bone marrow cells by centrifugation at 13,000*g* for 2 minutes. Cortical bone was then stored at −80°C before RNA extraction. Total RNA was purified from cortical bone or cultured cells using TRIzol Reagent (Thermo Fisher Scientific) according to the manufacturer’s instructions. RNA was quantified using a NanoDrop instrument (Thermo Fisher Scientific), and RNA integrity was verified by resolution on 0.8% agarose gels. 500 ng (cortical bone) or 1–2 μg (cultured cells) of RNA was then used to synthesize cDNA using the High-Capacity cDNA Reverse Transcription kit (Thermo Fisher Scientific) according to the manufacturer’s directions. Transcript abundance in the cDNA was measured by quantitative PCR using TaqMan Universal PCR Master Mix (Thermo Fisher Scientific). The following TaqMan assays were used: *Tnfsf11* (Mm0041908_m1); *Cdkn2a* (Mm00494449_m1); *Cdkn1a* (Mm00432448_m1); *Tnf* (Mm00443258_m1); *Il1a* (Mm99999060_m1); *Il6* (Mm00446190_m1); *Gata4* (Mm00484689_m1); *TNFSF11* (Hs00243522_m1); *IL1A* (Hs00174092_m1); and *CDKN1A* (Hs00355782_m1). The following transcripts were used for normalization: *Mrps2* (forward, 5′-CCCAGGATGGCGACGAT-3′; reverse, 5′-CCGAATGCTGTAATGGCGTAT-3′; probe 5′-FAM-TCCAGAGCAGGATCC-NFQ-3′); *Actb* (Mm00607939_s1); *B2m* (Mm00437762_m1); *Rn18S* (4319413E); *Hprt* (Mm00446968_m1); and human *GAPDH* (Hs99999905_m1). Gene expression was calculated using the ΔCt method (35). For the results in Figure 2C, *Tnfsf11* transcript levels were normalized to each of 5 housekeeping genes (*Mrps2, Actb, B2m, Rn18S*, and *Hprt*), and the transcript level was calculated as the geometric mean of the 5 normalized values.

### Western blot.

Osteocyte-enriched femoral cortical bone was prepared as described above. Bone fragments were immediately frozen in liquid nitrogen and pulverized. Proteins were extracted with a buffer containing 20 mM Tris-HCL, 150 mM NaCl, 1% Triton X-100, protease inhibitor mixture, and phosphatase inhibitor cocktail (MilliporeSigma) on ice for 30 minutes and then stored at –80°C overnight. The protein concentration of the bone extract was determined using the DC Protein Assay Kit (Bio-Rad). The extracted protein (20–30 μg per sample) was separated on 10%–15% SDS-PAGE gels and transferred electrophoretically onto PVDF membranes. The membranes were blocked in 5% fat-free milk/Tris-buffered saline for 120 minutes and incubated with a primary antibody, followed by a secondary antibody conjugated with horseradish peroxidase. Mouse monoclonal antibodies against g-H2AX (Millipore, 05-636, 1:5000), mouse monoclonal antibody for p16 (Santa Cruz Biotechnology, sc-1661, 1:2000), goat polyclonal antibody for GATA4 (Santa Cruz Biotechnology, sc-1237, 1:500), goat polyclonal antibody against RANKL (R&D Systems, AF462, 1:1000), and rabbit polyclonal antibody for p-p65 (Cell Signaling Technology, 3039, 1:1000) were used to detect their corresponding protein. Blots were stripped and reprobed with anti–β-actin antibody (Santa Cruz Biotechnology, sc-81178, 1:2000). Bound antibodies were detected with ECL reagents (Millipore) and imaged and quantified with a VersaDoc imaging system (Bio-Rad). Uncut gels are shown in Supplemental Figure 1 (supplemental material available online with this article; https://doi.org/10.1172/jci.insight.138815DS1).

### Retroviral infection.

A cDNA encoding full-length murine Gata4 was provided by Mona Nemer (University of Ottawa, Ontario, Canada) and subcloned into the pMX-IRES-EGFP retrovirus vector (Cell Biolabs Inc., RTV-013). The resulting construct was verified by DNA sequencing. Retroviral particles were generated by transfecting Plat-E viral packaging cells with the above-generated construct using Lipofectamine Transfection Reagent (Thermo Fisher Scientific). Supernatants containing viral particles were collected 48 hours after transfection, filtered through a 0.2 μm filter, and immediately used to transduce bone marrow stromal cells. The newborn calvaria cells or stromal cells were seeded in 10 cm culture dishes as described above and transduced with retroviral particles in α-MEM complete media with 8 μg/mL polybrene for 24 hours. Transduced cells were then selected by incubation with 1.5 μg/mL puromycin and then replated in 12- or 96-well tissue culture plates for subsequent analysis.

### BrdU assay.

Cell proliferation was quantified by incorporation of 5-bromo-2′-deoxyuridine (BrdU) into newly synthesized DNA. Cultured bone marrow stromal cells were washed with 1× PBS and subjected to a BrdU detection assay according to the manufacturer’s protocol (Roche Diagnostics).

### Caspase-3 activity.

Bone marrow stromal cells were lysed in 20 mM Tris-HCl (pH 7.5), 150 mM NaCl, 1 mM EDTA, 10 mM NaF, 1 mM sodium orthovanadate, 5 μg/mL leupeptin, 0.14 U/mL aprotinin, 1 mM phenylmethylsulfonyl fluoride, and 1% Triton X-100. Cell lysates were incubated with 50 mM DEVD-AFC in 50 mM HEPES (pH 7.4), 100 mM NaCl, 0.1% CHAPS, 10 mM DTT, 1 mM EDTA, and 10% glycerol. Caspase-3 activity was measured by quantifying the degradation of the fluorometric substrate DEVD-AFC (Biomol Research Labs). The released fluorescent product was measured in a microplate fluorescence reader with excitation/emission wavelengths of 340/542 nm. Total protein concentration was measured using the DC Protein Assay (Bio-Rad).

### Statistics.

For spine and femur BMD data in Figure 1A, repeated-measures ANOVA models were considered with main effects of genotype and month, interactions of the main effects, month as categorical or continuous variables, with a possible quadratic term. After examining significance of effects and model fit statistics, models with categorical effects of genotype, month, and their interaction were chosen for both models. These analyses were done with SAS v9.4. All other data were analyzed using Prism 8 (GraphPad Software). Two-way ANOVA was used to detect statistically significant treatment effects after determining that the data were normally distributed and exhibited equivalent variances. In some cases, log or ranks transformations were used to obtain normally distributed data and equal variance. This was followed by all pairwise comparisons using Tukey’s procedure. For experiments involving comparison of only 2 groups, a 2-tailed Student’s *t* test was used. A *P* value of less than 0.05 was considered significant.

### Study approval.

The Institutional Animal Care and Use Committee of the University of Arkansas for Medical Sciences reviewed and approved all studies involving mice.

## Author contributions

MA, CAO, JX, and HNK designed the experiments. HNK, JX, YE, EF, RSM, SI, KMC, and LH performed the experiments. HNK, JX, YE, RLJ, DZ, YF, JDT, MA, and CAO analyzed the results. MA and CAO wrote the first draft of the manuscript. All authors edited the manuscript.

## Supplementary Material

Supplemental data

## Version 1 09/01/2020

In-Press Preview

## Version 2 10/02/2020

Electronic publication
