# Supplementary figures and images for: Osteocyte RANKL is required for cortical bone loss with age and is induced by senescence
Source: JCI Insight. 2020 Oct 2;5(19):e138815. doi: 10.1172/jci.insight.138815 (PMC7566701; doi:10.1172/jci.insight.138815)

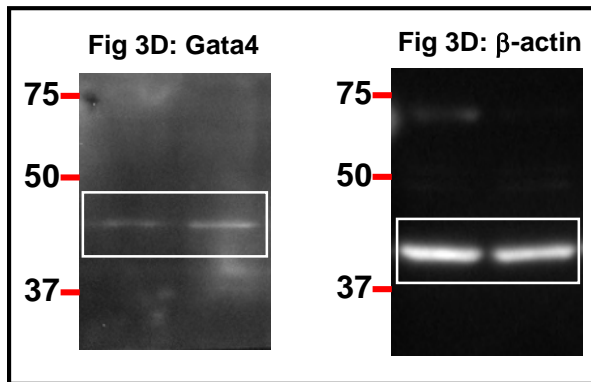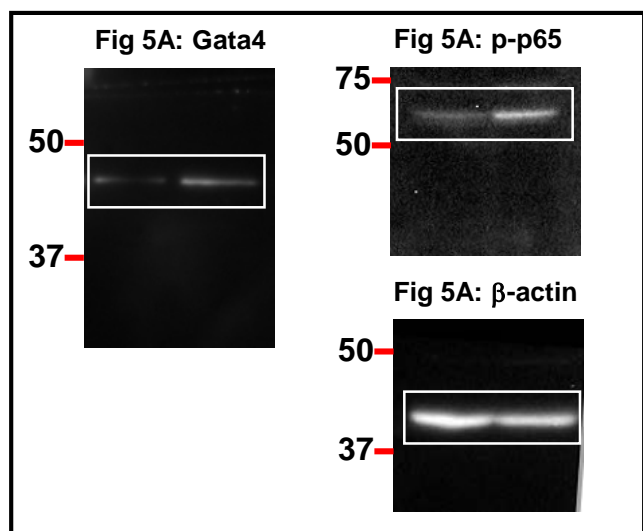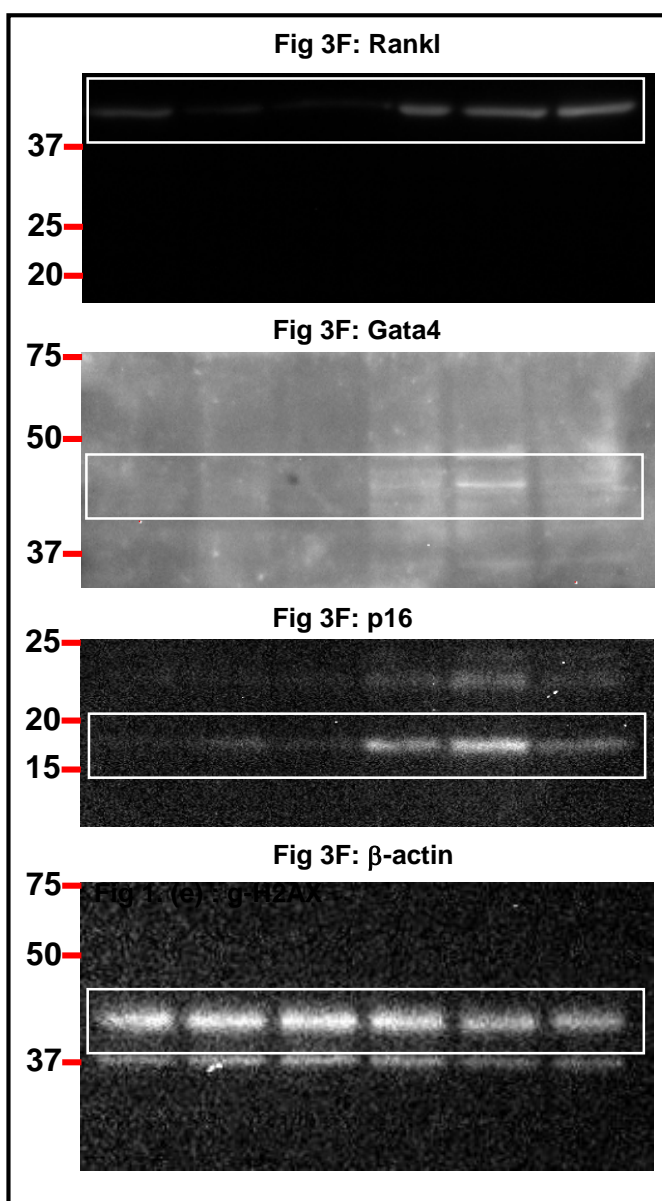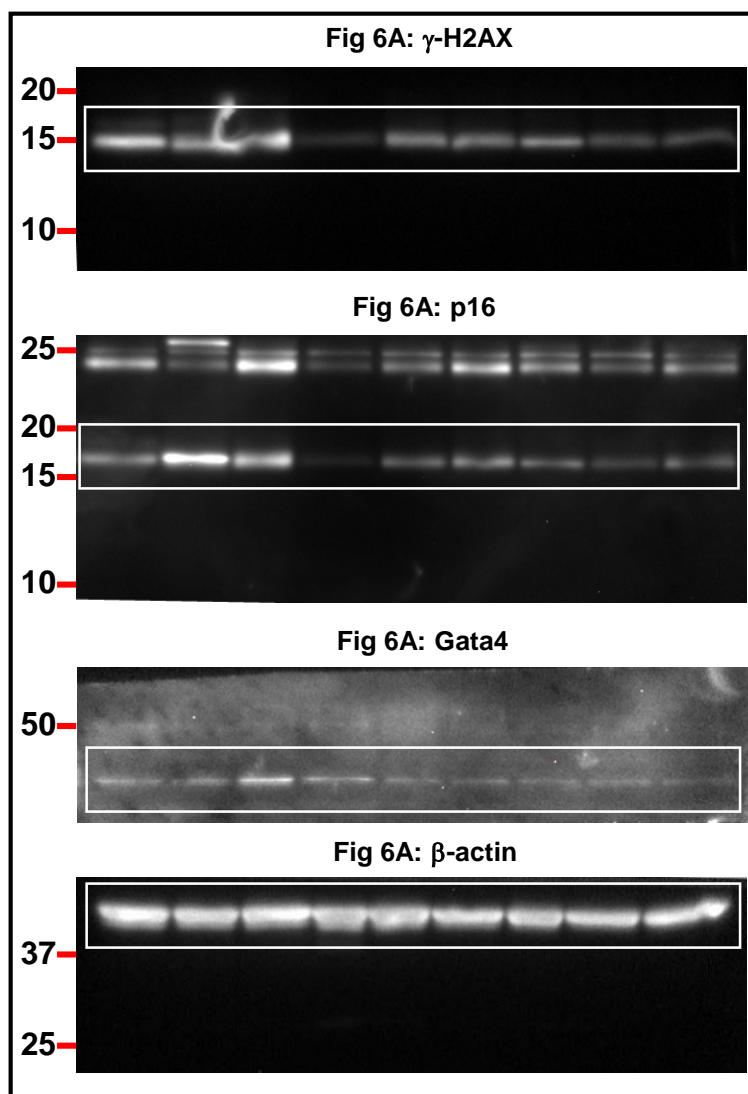

**Supplementary  
Figure**

Supplement: Supplemental data [file jciinsight-5-138815-s181.pdf]
